# Vision-Based Human–Robot Handover System with Reinforcement Learning

**DOI:** 10.3390/s26123811

**Published:** 2026-06-15

**Authors:** Weiliang Cao, Zhenwei Cao, Yong Song

**Affiliations:** 1 School of Engineering, Swinburne University of Technology, Melbourne, VIC 3122, Australia; weiliangcao@swin.edu.au; 2 School of Airspace Science and Engineering, Shandong University, Weihai 264209, China; songyong@sdu.edu.cn

**Keywords:** handover, human–robot, vision-based, simulation, collaboration

## Abstract

Handover control in human–robot collaboration remains a significant challenge. This paper proposes a three-step vision-based human–robot handover system (VHS). Vision inputs are used to perceive the environment and enable adaptive control of the robotic arm. Moreover, a three-step behavior cloning learning strategy is designed. Furthermore, a modified Temporal Difference (TD) loss function based on transfer models is proposed to train the algorithm to improve policy exploration and convergence. The proposed method results in substantial enhancements in comparative experimental validation in a simulation environment with a realistic dynamic hand model.

## 1. Introduction

Handover plays an important role in human–robot interaction and attracts widespread attention in recent research. It has a very broad application prospect in scenarios involving direct human–robot interaction, such as collaborative assembly tasks in industrial settings, housekeeping tasks, etc. There are numerous challenges in environmental presentation, control policy construction, safety assurance, etc. Unlike the top-down grasping task, handover requires generating a continuous control policy that transforms simple image input into complex interaction control strategies.

Vision-Based Human–Robot Handover.In vision-based human–robot handover tasks, visual inputs are used to perceive information about the hand, object and environment, thereby enabling the robot to execute the handover action [1]. In recent years, large-scale hand–object interaction datasets and advances in hand and object pose estimation techniques have lead to significant progress [2,3]. These developments facilitate model-based human–robot handover tasks through complete pose estimation and tracking. However, such methods rely on fixed object models and cannot generalize to unseen objects [4]. To address this issue, current research is shifting towards learning-based methods. These approaches directly learn grasping strategies from visual inputs (such as images or point clouds), enabling generalization to unseen objects. However, most of these methods are open-loop without feedback functions, which limits their ability to plan for dynamic scenarios [5]. Therefore, achieving end-to-end closed-loop real-time motion control strategies has become critical. In recent years, several DRL methods have demonstrated promising results [6,7]. However, further improvements in visual perception techniques and algorithm design are still needed.

Policy Learning and Algorithms. In robot grasping and handover tasks, traditional approaches rely on known object shape or pose to generate reliable grasp poses. However, extracting 3D object geometry information from real-world sensory inputs is challenging [3]. The latest research is shifting towards predicting grasp poses directly from sensor data using deep neural networks [5]. However, many of these methods decouple grasp prediction from trajectory planning, which can compromise stability, particularly in dynamic environments such as human–robot handover. To address these challenges, end-to-end learning approaches are being developed to learn closed-loop control policies directly from sensor data [1]. Recent studies have proposed many solutions, like self-supervised reinforcement learning (RL) methods, to learn deep Q-functions from real-world grasping demonstrations [8]. Furthermore, the combination of imitation learning and reinforcement learning algorithms is increasingly employed to generate grasping and handover strategies in dynamic environments [6]. These approaches facilitate more stable 6-DoF closed-loop control for grasping and handovers. However, there remains significant challenges in the design and optimization of policy learning, as well as in current algorithms.

Although most prior work focuses on top-down grasping strategies [9,10,11,12,13], recent efforts also investigate 6D robotic grasping, achieving notable progress [14,15,16]. The training process for such complex strategies is commonly performed in simulation environments, where image input is rendered into action interactions, and this safe and efficient approach is emerging as a new trend [15,17,18]. In recent years, a large amount of research in the field of robotic manipulation has yielded significant results based on these foundations. However, in the context of human–robot interaction, the primary difficulty lies in characterizing human behavior in simulation environments and enabling robots to learn through human–robot interactions [19,20,21]. Human agents are complex, evolving inputs to the environment that pose additional challenges. However, extensive work is being done to embed human behaviors as agents in simulation environments.

In a recent study, some scholars build a simulation environment for human–computer interaction and use a large amount of real human motion data to train the motion of virtual hands in the simulation environment [1], realizing interaction in the virtual environment and serving as a foundation for comparative research and benchmarking. Another work [6] studies the handover task using point cloud data on this basis and achieves good results. However, this method performs poorly with image input which has less information than cloud data.

The objective of this paper is to achieve a vision-based efficient human–robot handover system. The proposed approach builds on recent advances in vision-based human–robot handover tasks but does not rely on point cloud data. Instead, this work uses raw image input for perception. Specifically, for human hand information, this work uses the HandTailor [22] model, a monocular 3D hand recovery algorithm proposed in [22], to directly extract detailed hand-pose features from the image input. To better extract detailed features, DenseNet121 is used as the backbone network of the model. This is a common deep convolutional network architecture that can extract rich detailed information from visual data. In addition, SAC is utilized as a stable and sample-efficient algorithm in continuous control tasks. This work also introduces a transition model as a world model. To further enhance policy training, a newly designed TD loss is proposed to integrate this transition model into the learning process, thereby improving the robot’s temporal consistency and decision-making ability. To evaluate the proposed method, simulation tests are conducted to verify the model’s dynamic response to the human hand’s motion state and to ensure that the object can be successfully handed over. This paper presents a three-step vision-based human–robot handover system (VHS). The specific contributions can be summarized as follows:

(1) A vision-based system with the HandTailor module is proposed, which improves the ability of feature extraction capability. This perception module extracts sufficient features from the visual input, given limited information, to enable complex robotic arm control in unpredictable environments.
(2) A three-step SAC method is designed. This three-step SAC training method uses behavior cloning to ensure stable training. This method realizes training acceleration from the beginning and avoids training collapse.
(3) A modified TD loss with self-supervision is proposed to incentivize contact and accelerate training. The modified TD loss enhances the effectiveness of the proposed method by guiding the policy to explore better policies with a better understanding of the relationship between states.
(4) The simulation validation using the HandoverSim benchmark [7] with PyBullet shows that the proposed approach performs better in the handover scenarios.

The following sections introduce the proposed handover system, which includes vision-based environment perception, three-step SAC training, and a modified TD loss with self-supervision and simulation validation in a PyBullet environment.

## 2. The Proposed Vision-Based Human–Robot Handover System

**Figure 1:**
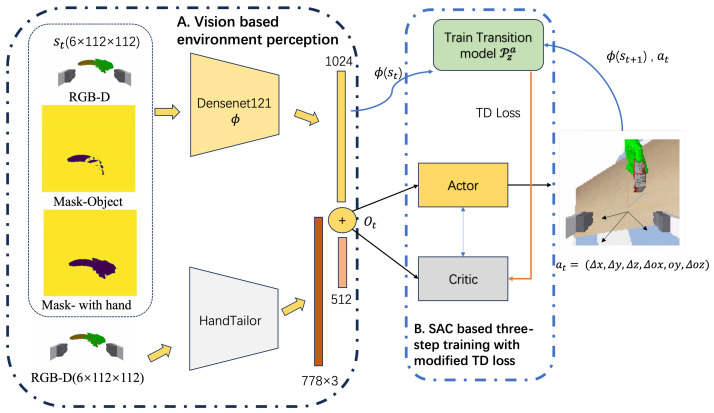
The overview of the proposed vision-based human–robot handover system (VHS).

The overall system is shown in Figure 1. The perception module comprises DenseNet 121 and HandTailor, which extract features from the visual input. The features are connected and sent to the SAC module, along with a new TD loss based on the transfer model, to generate an action $a_{t}\left(\Delta x,\Delta y,\Delta z,\Delta ox,\Delta oy,\Delta oz\right)$. The new TD loss based on the transfer model is used in the update process of the SAC algorithm module.

This reinforcement learning (RL) process for training the handover controller is a Markov Decision Process (MDP), defined as a tuple (S,A,P,R,γ), in which S is the state space, A is the action space, P is the state transition probability, R is the reward function, and γ∈[0,1] is the discount factor. According to [1], the handover task is divided into the approaching stage and the grasping stage. The grasping phase is judged by a trained grasp predictor and is only executed when it is close enough and the grasp confidence is very high; therefore, this work mainly focuses on the approach phase. Firstly, the visual information is captured by the RGB-D camera mounted on the end of the robot arm, and the perceptual information is transmitted to the policy π(a∣s) to give the action $a_{t}$, which represents the 6 -DOF pose (including translation and rotation) of the end effector in the next step. The pose of the end effector is translated into the target configuration of the robot kinematically, and then the joint torques are calculated and applied by the PD controller.

### 2.1. Vision-Based Environment Perception

The function of the perception module is to extract the features of the image input with two data streams concatenating the extracted features. The first part is the input of the RBG-D (4,112,112) image captured by the RGB-D camera at the end of the robot arm. The mask of the target object and the mask of the target object with the human hand are obtained using segmentation image technology as the other 2 dimensions, and the state $S_{t}$ of (6,112,112) is completed. The DenseNet 121 module is used for feature extraction. The 1024-long feature vector is extracted. To highlight the information of objects and hand models, the second part is designed to directly extract a feature mask of the human hand. The HandTailor framework [22] is used to detect hand models. HandTailor is chosen over MANO-based methods primarily for its efficiency and robustness in monocular settings. Compared to MANO-based approaches that often require iterative fitting, HandTailor provides a more direct and stable solution, making it better suited for real-time handover tasks. The HandTailor module includes a CNN-based hand grid generation module (hand module) and an optimization-based clipping module. The mano (the hand model used in HandTailor) grid is reconstructed by estimating the beta and theta parameters of the mano model [22]. For this part, the input is RBG-DDD (6,112,112). The output of the HandTailor model is a mesh data (778,3) to present accurate information, including position, shape, size, etc. Then, a three-layer fully connected multilayer perceptron is used to map the mesh data to a 512-dimensional feature vector. Finally, the outputs of these two parts are concatenated and sent to the control policy π.

DenseNet121 is used as the primary feature extractor in the perception module, capturing global visual information, including object appearance and scene context, from RGB-D inputs. This component acts as the backbone of the system and allows the policy to function even without additional hand-specific modeling. HandTailor is further introduced as an auxiliary module to provide 3D hand pose information. Although it cannot operate on its own in this framework, it provides the representation by adding structured information about hand configuration, which helps the model better capture hand–object spatial relationships.

As illustrated above, π(a∣s) is the actor network which is used to output action $a_{t}\left(\Delta x,\Delta y,\Delta z,\Delta ox,oy,\Delta oz\right)$. It takes the concatenated features from the DenseNet121 and HandTailor module as the input state $O_{t}$ for the Actor–Critic algorithm.

### 2.2. Three-Step SAC Training

The reinforcement learning framework is built upon the Soft Actor–Critic (SAC) algorithm. Compared with the DDPG-based method used in [6], SAC [23] introduces an entropy regularization term, which improves exploration and training stability in continuous control tasks. SAC algorithms are designed to learn a policy that is both highly rewarding and exploratory, thereby achieving better generalization in unknown environments. In the SAC algorithm, the entropy temperature parameter α plays a crucial role in controlling the policy’s entropy, thereby affecting the trade-off between exploration and exploitation. A larger α tends to increase the entropy of the policy, promoting more exploration; a smaller α, on the other hand, reduces exploration and makes the policy more likely to leverage existing experience. According to [24], a mechanism that automatically adjusts the entropy coefficient is used.

Unlike previous work, a three-step behavior cloning training process is proposed. Although the entire framework operates on visual observations, directly training a policy is still challenging due to perception noise and the increased difficulty in dynamic interaction scenarios. The proposed method trains three policies progressively. Generally, the first and second stages are trained with ‘static’ mode in which the robotic arm conducts actions after the human hand reaches static state. The third stage is training with the human hand in motion.

In the first step, an initial policy $\pi_{o}$ is trained using the visual input without the HandTailor model. The baseline policy is an existing work training with the human hand in ‘static’ mode which provides [6]. The behavior cloning in this step accelerates the training speed. This stage aims to learn stable approaching behavior while avoiding the instability introduced by hand pose estimation. The learned policy $\pi_{o}$ serves as a baseline policy for subsequent training.

In the second step, $\pi_{o}$ serves as the baseline policy. The network weights of $\pi_{o}$ are frozen at this stage. HandTailor model engages in this step to train the proposed policy $\pi_{s}$. This training step enables the proposed policy $\pi_{s}$ to learn more information from the vision input and to mitigate the unstable effects of the HandTailor model caused by the absence of a human hand in the view field. The policy is trained to be closer to the policy without using the HandTailor model to enhance the stability.

In the third step, the policy $\pi_{s}$ trained in step two is adopted as the baseline policy, with the network weights frozen. During this step, the policy $\pi_{d\;}$ is trained in the ‘dynamic’ mode in which the hand model is moving. This setting is used to keep the policy close to the policy with the ‘static’ mode.

Consequently, there are three policies: $\pi_{o}$ is a vision-based policy without the HandTailor model. $\pi_{s}$ incorporates hand pose information. $\pi_{d\;}$ is trained for dynamic handover scenarios. The loss function $L_{1}$ for training the actor in the first step policy $\pi_{o}$ is shown as follows:

(1) $$L_{1}=\mu L_{bc1}+\left(1-\mu \right)L_{SAC}$$

where $L_{bc1}$ is a loss of behavior cloning for training the policy closer to [6]. $L_{SAC}$ is the loss function to maximize the Q-value $\left(E\left[Q\left(s_{t},a_{t}\right)|a_{t}=\pi \left(s_{t}\right)\right]\right)$. μ is the control parameter to control the relationship between behavior cloning and the reinforcement learning training process. The loss function $L_{2}$ for training the actor in the second step policy $\pi_{s}$ is illustrated as follows:

(2) $$L_{2}=\lambda L_{bc1}+\left(\mu -\lambda \right)L_{bc2}+\left(1-\mu \right)L_{SAC}$$

where $L_{bc2}$ is the loss of behavior cloning to training the policy closer to $\pi_{o}$. λ and μ are used to balance these two behavior cloning and the reinforcement learning objectives. The lose function $L_{3}$ for training the final policy $\pi_{d\;}$ is presented as follows:

(3) $$L_{3}=\mu L_{bc3}+\left(1-\mu \right)L_{SAC}$$

where $L_{bc3}$ is the behavior cloning loss for training the policy closer to $\pi_{s}$. μ is used to balance these two learning objectives as above.

The TD loss is used as a basic loss function for the critic updating. In the next section, a novel modified TD loss is proposed.

As shown in Table 1, the most important hyperparameter are shown. Among them, the value of μ and λ are chosen by conducting a sensitive test. However, these two parameters are not the main contribution to this work and are not sensitive to the result.

### 2.3. Modified TD Loss with Self-Supervision

Sparse rewards are used to train the SAC algorithm in this environment. Rewards are only given if the mission is successful. Since the reward is zero most of the time, the agent can learn undesirable behaviors, such as staying in a certain state instead of actively trying to complete the task.

**Figure 2:**
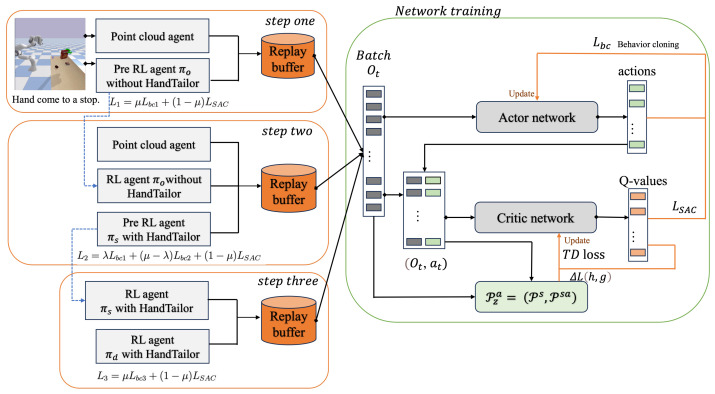
The block diagram of the proposed three-step SAC training system. Loss of behavior cloning is different corresponding to each training step as illustrated above. Point cloud agent is a point cloud based handover agent proposed in [6]. $P_{z}^{a}$ is the transition model training separately by self-supervision.

As shown in Figure 2, a new TD loss designed with the transition model is used to encourage the agent to explore more valuable action during the training process with the sparse-reward problem.

(4) $$P_{Z}^{a}:=\left(P^{s},P^{sa}\right)$$

Inspired by [25], a transition model $P_{z}^{a}$ is built to perform state-action representation learning, where there are two encoders:

(5) $$P^{s}=h\left(s\right),P^{sa}=g\left(P^{s},a\right)$$

$P^{s}$ individually encodes the state. $P^{sa}$ encodes both the state and the action. Both are represented in practical applications using a fully connected three-layer neural network. To train them, the following loss function [25] is used:

(6) $$L\left(h,g\right)={\left(g\left(h\left(s\right),a\right)-\left|h\left(s^{\prime }\right)\right|\right)}^{2}={\left(P^{sa}-\left|P^{s\prime }\right|\right)}^{2}$$

where $s^{\prime }$ is the next state and, according to [25], a state-action representation embedding is realized. The input of the *Q* network changes $\left(s_{t},a_{t}\right)\to \left(s_{t},a_{t},P^{s},P^{sa}\right)$, and for π will change $\left(s_{t}\right)\to \left(s_{t},P^{s}\right)$.

Unlike [25], $P_{z}^{a}$ is used as a world model here to indicate a new TD loss for updating the critic:

(7) $$TD_{loss}=E\left[\left(r\left(s_{t},a_{t}\right)+\gamma \cdot V\left(s_{t+1}\right)-Q\left(s_{t},a_{t},P^{s},P^{sa}\right)\right)+\beta \cdot \Delta L\left(h,g\right)\cdot r\left(s_{t},a_{t}\right)\right]$$

where β is a control parameter and set to 0.1. The modified TD loss in Equation (7) introduces $\beta \cdot \Delta L\left(h,g\right)\cdot r\left(s_{t},a_{t}\right)$, which provides exploration ability and regularization of consistency with the transition model. ΔL(h,g) is defined as the temporal difference of the transition model loss between two consecutive time steps:

(8) $$\Delta L\left(h,g\right)=L{\left(h,g\right)}_{t}-L{\left(h,g\right)}_{t-1}$$

where $L{\left(h,g\right)}_{t}$ denotes the transition model loss at time step *t*. This formulation approximates the temporal variation of the prediction error in a discrete-time setting. ΔL(h,g) reflects how the prediction error evolves over time. A larger value indicates greater uncertainty or novelty in the current state–action transition, which can be used as an auxiliary signal to enhance exploration during policy learning. When ΔL(h,g)<0, ΔL(h,g)=0; when ΔL(h,g)>10, ΔL(h,g)=10. Therefore, ΔL(h,g) is bounded and belongs to [0, 10] and since ΔL(h,g) is a transfer model, its result approaches 0 with continuous training. Therefore, it will not cause unstable numerical fluctuations in $TD_{\mathrm{loss}\;}$ and affect its convergence. Because it is reasonable to give an exploration reward when the current action explores the new state-action correspondence, it meets the requirement of reasonable guidance convergence.

## 3. Simulation Validation with PyBullet

### 3.1. Simulation Environment

**Figure 3:**
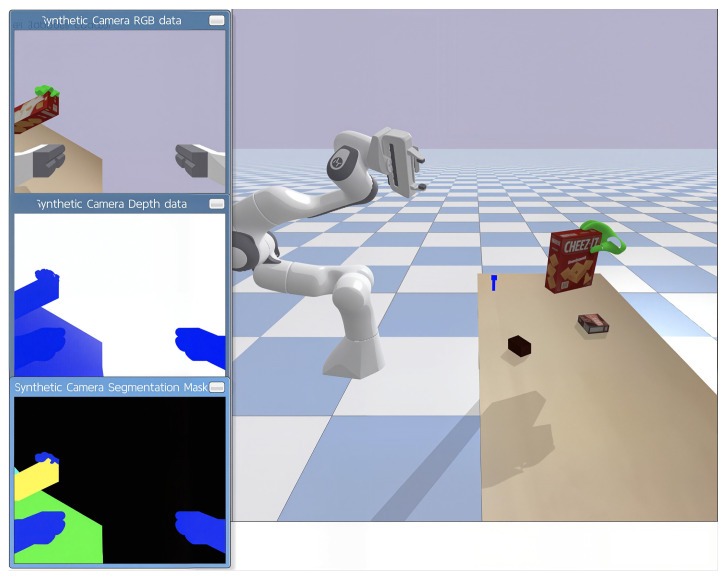
Simulation environment for the handover task in PyBullet.

HandoverSim [7] provides more than 1000 scenes with unique train, validation, and test sets. As shown in Figure 3, the simulation environment involves a human hand, a panda robotic arm, and an eye-in-hand camera. The robot interacts with a human hand to handover an object. The setup includes diverse objects and dynamic hand motions to simulate realistic interaction scenarios. There are two settings; the first ‘static’ mode means that the robotic arm can only move when the human hand reaches the target position. The second ‘dynamic’ mode means that the robotic arm can move in sync with the human hand. Ref. [7] also provides several evaluation metrics. Successful handover is defined as the success of grasping the target object without contact with the human hand, dropping the object, and where the use time exceeds the maximum limit (time out). For comparison, the baselines used here include GA-DDPG [1] and OMG Planner [26], both of which are solid works.

The simulation environment is based on the PyBullet physics engine. Panda is a single-joint, seven-axis robotic arm with a torque sensor that is introduced by Franka Emika GmbH (Munich, Germany). It is widely used in many industries, including logistics and warehousing, scientific research, and automated manufacturing. PID control is employed and is modeled in the physics engine.

Numerous control modes, including joint space position control, joint space torque control, Cartesian space pose control, and others, are supported by the Panda robotic arm’s control system. A description of the procedure for using spatial joint position control in the simulation environment is required. The angle of each joint in the Panda robotic arm is typically represented as a seven-dimensional vector. The robot arm has two fingers for grasping items, in addition to its seven joint angles, making its state nine-dimensional; this also includes the status of two parallel grippers. An RGBD camera at the robotic arm’s end captures color images in three dimensions and depth data about the object it is pointing at.

**Figure 4:**
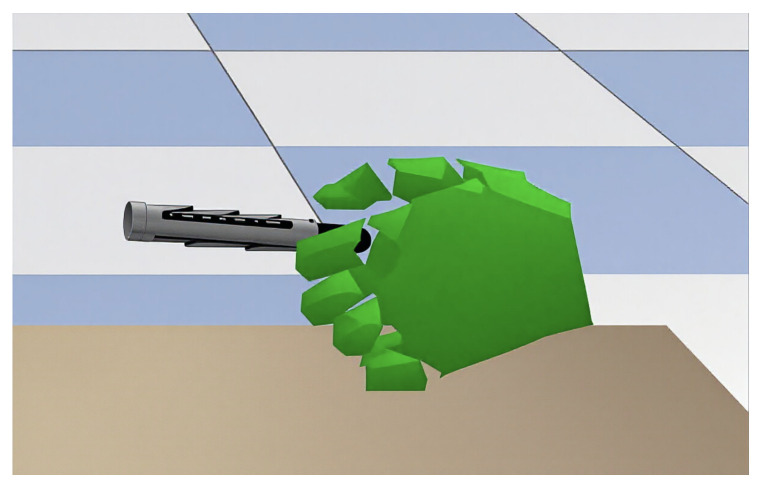
The rigid body model of human hand joints in PyBullet.

The PyBullet mano project in the PyBullet physics engine is used to import the preprocessed human hand joint rigid-body model in Figure 4. The hand motions are captured through recording real humans holing objects. The human hand model is divided into 16 links (links) and passed through the corresponding joints (joints) to connect. The model is described as an urdf file. In this way, the position and joint changes of the human hand model can be stored directly, without replaying all the mesh grids. The modeled human hand picture is shown in the Figure 4:

The simulation scenario is constructed in PyBullet in which the robotic arm performs the handover task between the human hand and the robotic arm. The robotic arm is controlled to grasp the object handed by the human hand model without touching the human hand. The 6D pose of each object in the YCB-video dataset comprises the object’s translation (x, y, z) and rotation (roll, pitch, yaw), describing the 3D position and orientation of the end effector.

The benchmark environment is built using the OpenAI Gym API and PyBullet. At each time step, the camera fixed on the end effector of the robotic arm captures the current state of the environment and passes it to the controller. The controller then generates an action based on its policy and performs it as $s_{t}\in S,a_{t}\in A$. After that, the environment is transferred to the new state $s_{t+1}$; at the same time, a scalar reward *r* is returned.

**Figure 5:**
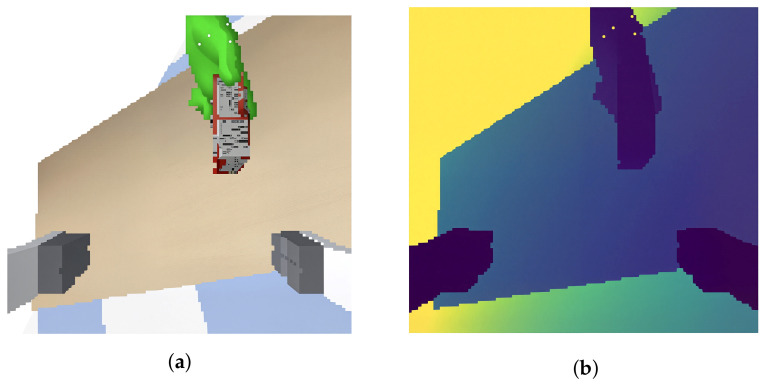
The camera RGB information (**a**) and the depth information (**b**).

The RGB observation picture and depth information captured by the camera are shown in Figure 5. The camera is fixed on the end-effector of the robotic arm.

In the simulation environment of this study, a more intuitive and natural action-space representation is adopted: the motion offset of the manipulator end-effector. The action space is a 6D continuous space, corresponding to the three translation components (x,y,z) and the three rotation components (roll, pitch, yaw) in the Cartesian coordinate system. Specifically, at each time step, the agent outputs a 6D action vector that represents the expected displacement and rotation changes of the manipulator’s end effector relative to its current position and attitude. This action vector is fed into an inverse kinematics solver, which converts the motion in Cartesian space into angle changes at the seven joints of the robotic arm to achieve the desired end motion $a_{t}=\left(\Delta x,\Delta y,\Delta z,\Delta ox,\Delta oy,\Delta oz\right)$. Using this terminal-motion offset as an action representation is more intuitive and natural, in line with humans’ natural way of understanding robot motion. At the same time, the dimensionality of the action space is reduced from the original nine dimensions (seven joint angles + two gripper states) to six dimensions, thereby reducing the difficulty of policy learning.

### 3.2. Validation of the Proposed Method and Ablation Analysis

**Figure 6:**
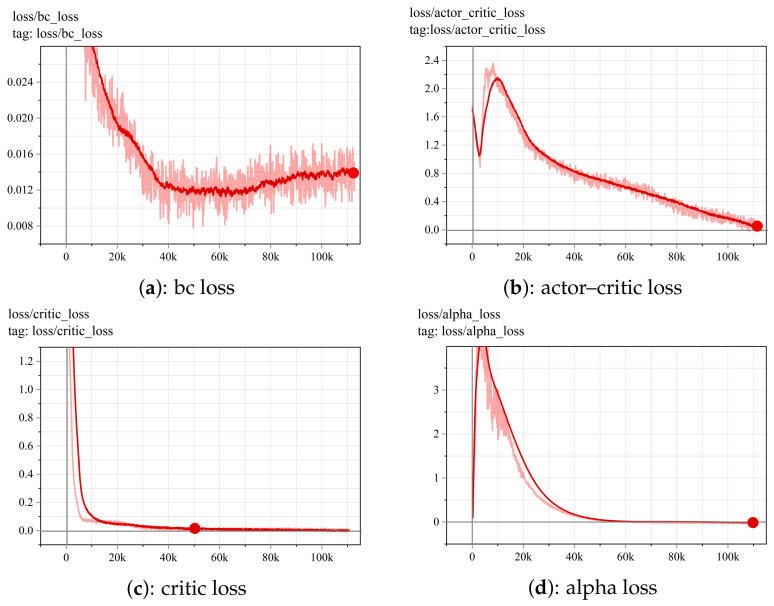
The four most important loss convergences during training.

As shown in Figure 6, the BC loss (step 3 here), actor loss, critic loss, and alpha loss all converge during training, supporting the validity and convergence of the proposed method.

As shown in Table 2, in ‘static’ mode, the human hand remains stationary and waits for handover from the moving robotic arm. All results are reported as mean ± standard deviation over three independent runs. The proposed method leads with a success rate of 70.52% and has the lowest contact rate (5.62%), demonstrating the best overall performance; the proposed method without HandTailor performed the best in the time out rate (6.51%), but has a higher contact rate (12.62%); OMG Planner [26] has the lowest drop rate (5.76%), but has a poor success rate (60.22%) and contact rate (26.34%); GA-DDPG [1] does not have outstanding indicators, except the highest drop rate (23.63%). The results indicate that the proposed method has significant advantages in task completion rate and operation safety.

In ‘dynamic’ mode, human hands move while the robotic arm approaches and grasps the object. All results are reported as mean ± standard deviation over three independent runs. According to Table 3, the success rate of the proposed method is 65.52%, which is 13.25% higher than that of GA-DDPG. It can be concluded that the proposed method has a higher execution efficiency in dynamic scenarios. The contact rate of the proposed method is 6.25%, which is lower than the 12.18% of GA-DDPG. This validates its advantage in reducing contact and effectively overcoming the baseline standard. The drop and time out rates of GA-DDPG are 24.72% and 10.83%, respectively, which are significantly higher than those of the proposed method. The proposed method has a higher success rate and a lower contact rate in both ‘static’ and ‘dynamic’ modes, especially in reducing the contact rate. These results show that the proposed method can effectively learn the influence of hand features on handover tasks and has good adaptability and efficiency in different scenarios.

#### 3.2.1. Ablation Analysis

**Figure 7:**
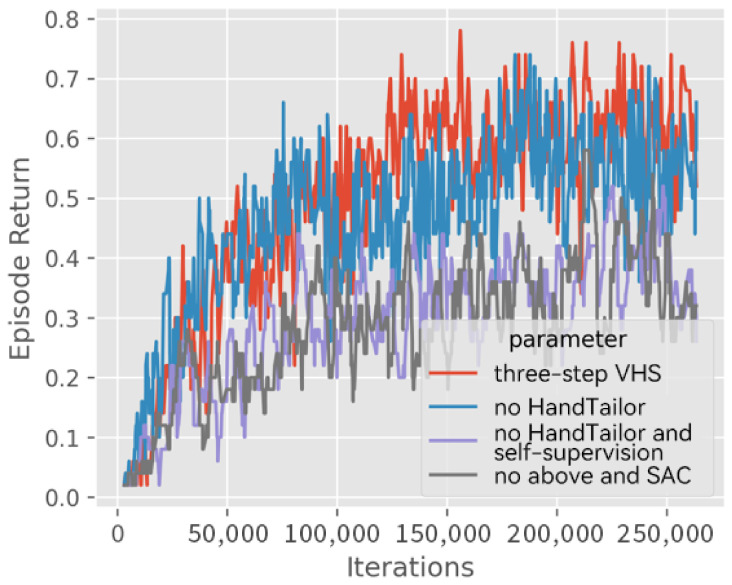
The ablation analysis of the proposed three-step vision-based human–robot handover system.

The reward for a handover episode is set as 0 for fail and 1 for success. The Episode Return represents the handover success rate in this iteration. As displayed in Figure 7, the ablation experiment shows that the perceptual mask of HandTailor and the newly designed self-supervision TD loss have a significant synergistic effect on the performance of the agent. The complete three-step vision-based human–robot handover system (VHS, red curve) shows the highest return value about 0.7. The blue curve shows the performance apparently decreases after removing the HandTailor model. The purple curve indicates the performance further decreases after removing the HandTailor model and the modified TD loss. The gray curve displays the lowest success rate after removing the HandTailor model, the modified TD loss, and the SAC entropy, which is DDPG. Figure 7 also highlights that the purple curve is similar to the grey curve, which means, without the proposed modification, DDPG and SAC perform similarly.

**Figure 8:**
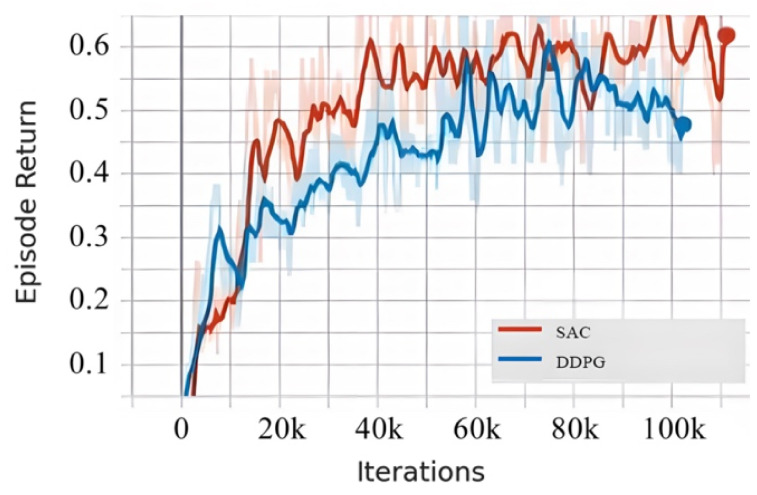
Comparison of the proposed system with SAC and DDPG. Episode Return means the success rate at corresponding iterations.

A comparison is conducted for the proposed modifications with SAC and DDPG algorithms. Figure 8 shows that dynamic entropy in SAC is essential, which has a higher Episode Return, in turn meaning a better success rate at corresponding iterations. DDPG shows excellent study ability at the beginning, while clearly SAC has a more stable and stronger learning ability with the dynamic entropy to explore better policy with the proposed methods.

#### 3.2.2. Domain Randomization

To evaluate the robustness of the proposed method, experiments under domain variation are conducted with different types of environment noises, including lighting changes, object variations, hand motion noise, and Gravity.

The results are summarized in Table 4. Compared to the proposed method without randomization, performance decrease with the environmental uncertainty increases. The proposed method is proved to have robustness and generalization capability to diverse conditions. This result improves sim-to-real transfer performance and demonstrates strong adaptability to changing environments.

This experiment setting in simulation has many similarities to real-world conditions. The object contains numerous categories, and the human hand motion is obtained human motion capture data. However, there are still some limitations in sim-to-real transfer, such as the absence of full human body models and the diversity in human hand appearances (color, size, etc.). In future, the proposed work can be conducted on physical robotic arms.

## 4. Summary

In this paper, a novel three-step vision-based human–robot handover system (VHS) is presented for human–robot handover tasks. An effective vision-based perception module is used to extract features of object and human hand poses, providing a robust capability for continuous control in unpredictable environments. In addition, a transition model is integrated with a modified TD loss in the proposed three-step SAC framework, thereby enhancing temporal consistency and decision-making of the control policy. The proposed approach demonstrates promising results in simulation tests, highlighting its potential to handle dynamic handover scenarios.

## Figures and Tables

**Table 1:** The most important hyperparameters.

Parameter	Value
General Training Settings	
Parallel workers	3
Simulation timestep	$1\times 10^{-3}$ s
Simulation steps per action	150
Optimizer	Adam
Activation function	ReLU
Replay Buffer	
Buffer size (Stage 1)	$1\times 10^{6}$
Buffer size (Stage 2)	$4\times 10^{5}$
Buffer size (Stage 3)	$4\times 10^{5}$
Three-Step Training	
Iterations (Stage 1)	10,000
Iterations (Stage 2)	5000
Iterations (Stage 3)	5000
μ (Stage 1)	0.3
μ (Stage 1)	0.4
μ (Stage 1)	0.3
λ (Stage 2)	0.1

**Table 2:** HandoverSim benchmark evaluation for ‘static’ mode (mean ± standard deviation over three independent runs).

Method	Success	Contact	Drop	Time Out
OMG Planner [26]	60.22 ± 1.8	26.34 ± 2.1	5.76 ± 0.9	7.68 ± 1.2
GA-DDPG [1]	55.31 ± 2.5	12.73 ± 1.6	23.63 ± 2.8	8.33 ± 1.5
Proposed w/o HandTailor	64.65 ± 1.9	12.62 ± 1.4	16.22 ± 2.0	6.51 ± 1.1
Proposed w/o modified TD loss	62.80 ± 2.1	13.95 ± 1.7	17.85 ± 2.3	7.10 ± 1.3
Proposed method	70.52 ± 1.6	5.62 ± 1.2	15.29 ± 1.8	8.57 ± 1.4

**Table 3:** HandoverSim benchmark evaluation for ‘dynamic’ mode (mean ± standard deviation over three independent runs).

Method	Success	Contact	Drop	Time Out
GA-DDPG [1]	52.27 ± 2.7	12.18 ± 1.9	24.72 ± 3.1	10.83 ± 1.6
Proposed method	65.52 ± 2.0	6.25 ± 1.3	19.51 ± 2.4	8.72 ± 1.5

**Table 4:** Evaluation under domain randomization settings (mean ± standard deviation over three independent runs).

Setting	Success	Contact	Drop	Time Out
Proposed w/o randomization	70.52 ± 1.6	5.62 ± 1.2	15.29 ± 1.8	8.57 ± 1.4
Lighting changes	68.10 ± 2.1	6.85 ± 1.5	16.92 ± 2.2	8.13 ± 1.6
Object variation	67.45 ± 2.3	7.10 ± 1.7	17.36 ± 2.5	8.09 ± 1.5
Hand motion noise	65.82 ± 2.6	8.25 ± 1.9	18.74 ± 2.8	7.19 ± 1.7
Gravity (1–2 times)	64.37 ± 2.8	8.96 ± 2.1	19.85 ± 3.0	6.82 ± 1.8
Full randomization	63.15 ± 3.0	9.52 ± 2.3	20.73 ± 3.2	6.60 ± 1.9

## Data Availability

The data that support the findings of this study are available from the corresponding author upon reasonable request.
